# Oral Self-Mutilation in Lesch-Nyhan Syndrome: A Case Report

**DOI:** 10.7759/cureus.27874

**Published:** 2022-08-11

**Authors:** José Ferrão, Cristina Rodrigues Barros, Luísa Figueiredo, Ana Fernandes

**Affiliations:** 1 Oral and Maxillofacial Surgery, Centro Hospitalar Universitário de Lisboa Central, Lisbon, PRT; 2 Pediatric Stomatology Department, Hospital Dona Estefânia - Centro Hospitalar Universitário de Lisboa Central, Lisbon, PRT

**Keywords:** dental care for children, self-mutilation, self-biting, lip injuries, lesch-nyhan syndrome

## Abstract

Lesch-Nyhan syndrome (LNS) is an inherited recessive X-related disorder caused by a deficiency of the purine salvage enzyme hypoxanthine-guanine phosphoribosyltransferase. It is characterized by dystonia and compulsive self-mutilation, in particular, biting behavior on the oral mucosa, tongue, lips, fingers, and shoulders, typically before one year of age. The majority of these patients require several procedures, including dental extractions, to prevent significant secondary lesions. This article aims to report a clinical case of a 12-year-old boy with an LNS diagnosis who was referred to the Paediatric Stomatology Department of Central Lisbon University Hospital. Since the age of eight, the patient had displayed self-harm behavior, with arm and oral injuries. On evaluation, he presented with deep ulcerated lesions on the lips and tongue, with substance loss associated with a significant decrease in food intake and consequent weight loss. The management included conservative therapy with gabapentin, lorazepam, and botulinum toxin injections. A successful reduction of self-mutilation with no signs of new lesions in the oral cavity and an improvement in nutritional status were reported. The therapeutic approach is essential to provide the best quality of life for patients and their caregivers. To delay radical treatments, multiple therapeutic options can be used. The oral pathology team considered that the most appropriate therapy was botulinum toxin A injections along with therapeutic adjustment, which was effective in wound healing and self-mutilation behavior ceasing at the two-month follow-up.

## Introduction

Lesch-Nyhan disease (LND), first described in 1964, is an X-linked recessive disorder caused by a deficiency in the purine salvage enzyme hypoxanthine-guanine phosphoribosyltransferase (HPRT) [1]. It affects 1:100,000 to 1:380,000 newborns, mostly males.

The deficiency in the HPRT activity leads to hyperuricemia and deposition of urate crystals in peripheral tissues. This causes neurological, renal, and musculoskeletal manifestations, such as developmental delay, growth and mental retardation, choreoathetoid spasticity, nephrolithiasis, obstructive nephropathy, and acute gouty arthritis. Death usually occurs in the second or third decade of life secondary to infection or renal failure [2].

The residual activity of the HPRT enzyme determines the clinical phenotype. The stage of compromise is classified as grade one, with no neurological manifestations, to grade four “classic Lesch-Nyhan syndrome,” with a complete deficiency in HPRT and acute generalized dystonia associated with cognitive impairment, megaloblastic anemia, ballism, choreoathetosis, and behavior alterations, including self-aggressive behaviors [3,4].

The abnormal movements and compulsive self-aggression normally occur before the first year of life and often present as the first symptoms of the disease. They are expressed as persistent bites in the oral mucosa, tongue, lips, fingers, and shoulders, leading to the total or partial destruction of the affected tissues. Mutilation is not due to a lack of sensation, but induced by an obsessive-compulsive behavior, with the child experiencing pain similar to the general population. This might be explained by reductions in the volume of the ventral striatum and absent prefrontal areas [5].

In some cases, self-mutilation with external surfaces, such as a wheelchair component, head banging, or extended arms in doorways, as instruments of self-injury, tend to co-occur [6].

## Case presentation

A 12-year-old boy with a previously biochemical and molecular diagnosis of LNS was referred to the Paediatric Stomatology Department, in Central Lisbon University Hospital (CHULC), for severe perioral and intraoral lesions.

In the context of his disease, the patient suffered from focal epilepsy and psychomotor development disorder. Hence, he usually presented with marked motor incoordination, hyperreflexia, hyperkinetic choreoathetoid movements, and self-harm behavior with autoinflicted lesions and ataxia, all of which were intensified by stress. For that reason, the child used a specially designed wheelchair, fitted with a seat belt to keep him upright, to prevent head injuries and uncontrolled movements.

While recording the medical history, it was assessed that the patient had developed a habit of self-biting at eight years of age, shortly after the onset of the eruption of permanent teeth.

**Figure 1:**
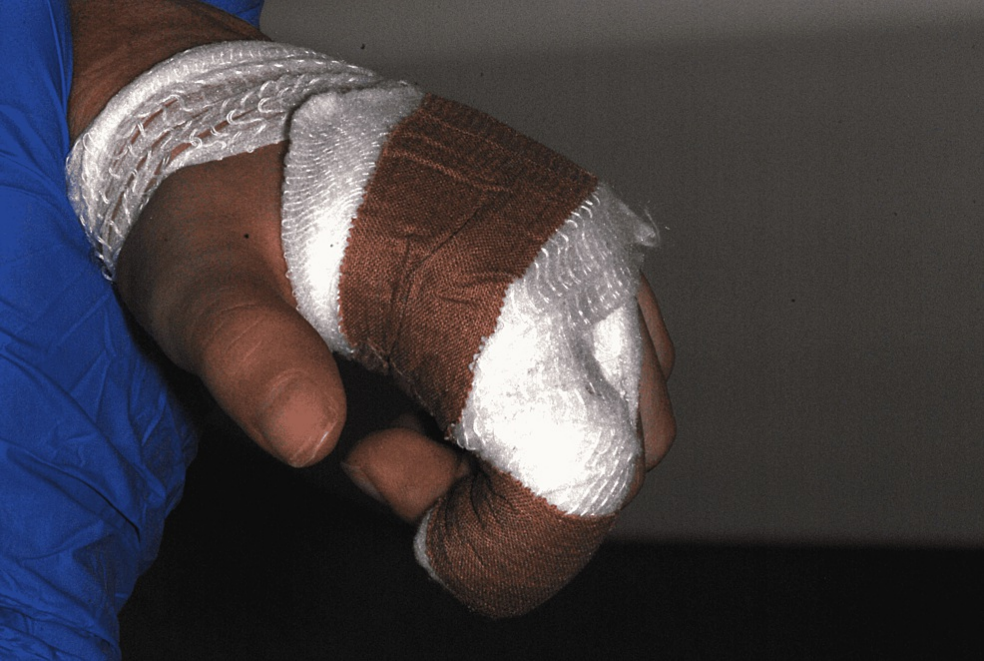
Wraps to prevent finger biting.

To control his symptoms, he was medicated but the response was fairly null. On presentation, a general physical examination showed the child with restrained arms with wraps to prevent finger biting (Figure 1).

**Figure 2:**
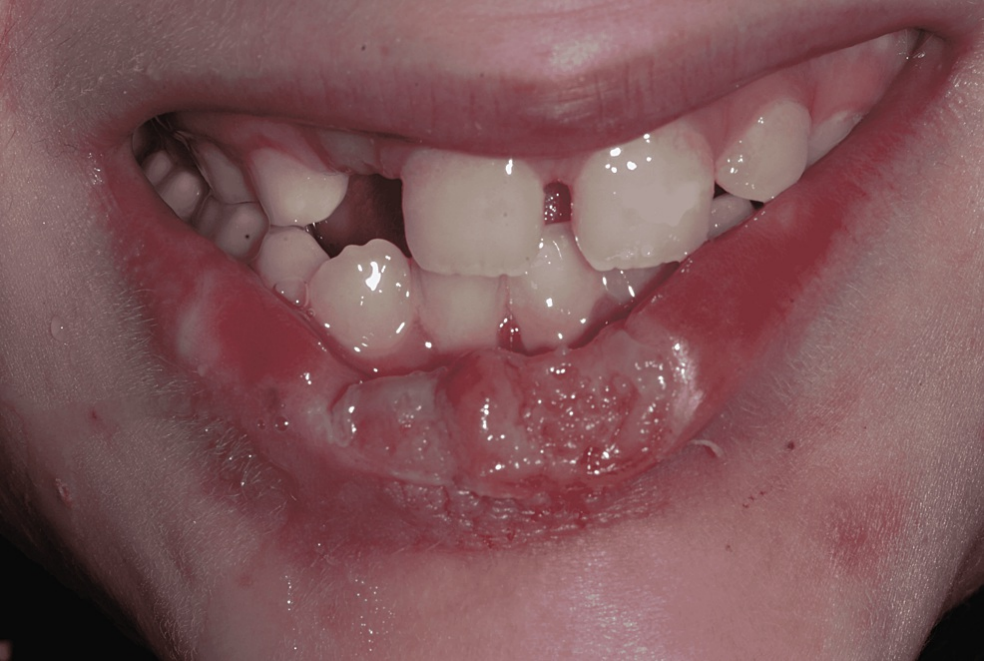
Large irregular ulcerative lesion, mouth opened.

**Figure 3:**
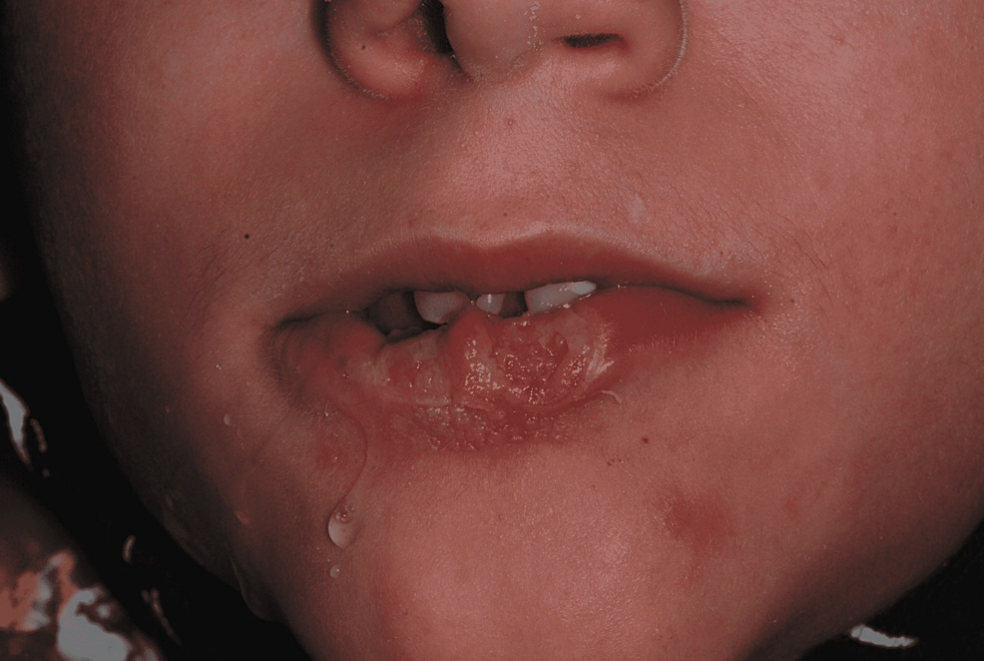
Large irregular ulcerative lesion, mouth closed.

Extraoral examination revealed a large irregular ulcerative lesion in the midline of the lower lip, with raised edges, and some regions covered by small layers of fibrin (Figures 2, 3). There were no signs of bleeding or suppuration. The surrounding lip area had a contused hyperemic wound.

**Figure 4:**
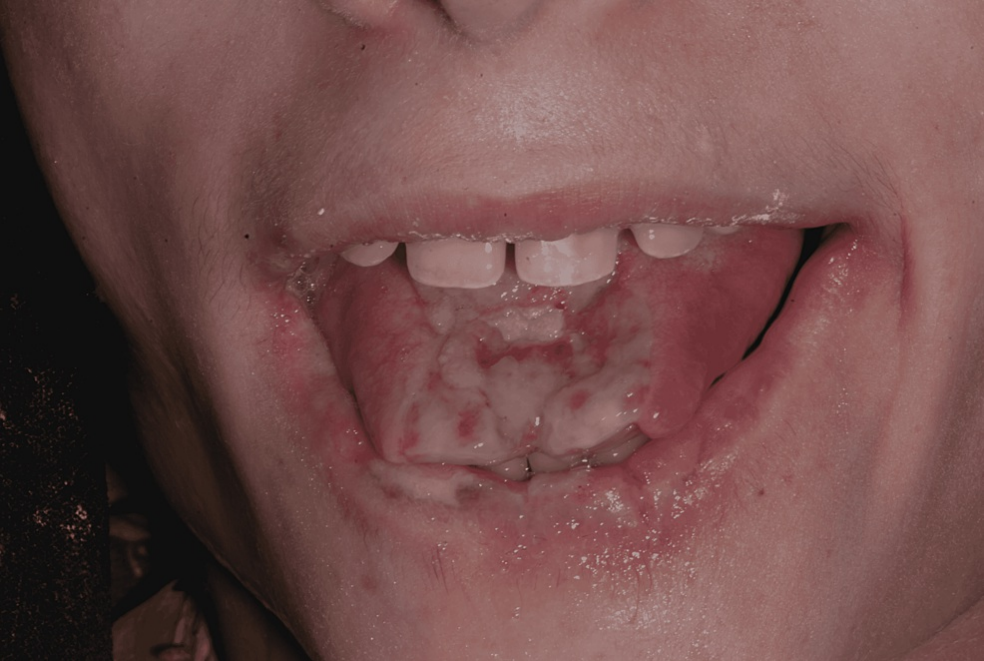
Tongue ulcer and apical substance loss.

On intraoral observation, the anterior dorsum of the tongue presented with a single, ill-defined, deep depressible, hemorrhagic, painless ulcer, caused by maxillary incisors, with loss of substance (Figure 4). The surrounding tissues of the tongue had associated inflamatory signs. No other lesions were seen in the remaining oral mucosa. The dentition was mixed and his oral hygiene was poor, with multiple dental caries.

The choice of the right therapeutic approach should consider the patient’s physical and cognitive capacities, self-care capacity, communication, degree of independence, and compulsive self-mutilating behavior. In collaboration with the Metabolic Diseases Department, a therapeutic adjustment was provided with gabapentin, 100 mg, three times daily, and lorazepam, 1 mg/24 hours, to control neuropsychiatric symptoms. Additionally, botulinum toxin A injections were administered into the bilateral masseters to reduce self-abusive behavior. The use of intraoral devices (anterior bite block) and multiple extractions of all erupted maxillary permanent incisors in the operating room and under general anesthesia were also considered.

**Figure 5:**
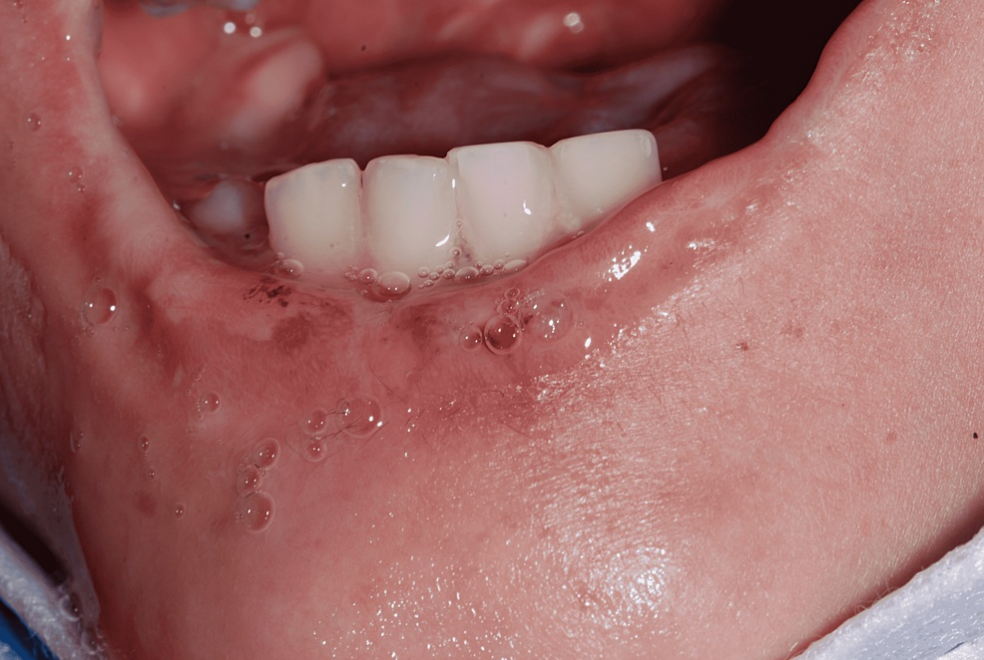
Lip scar at the two-month follow-up.

**Figure 6:**
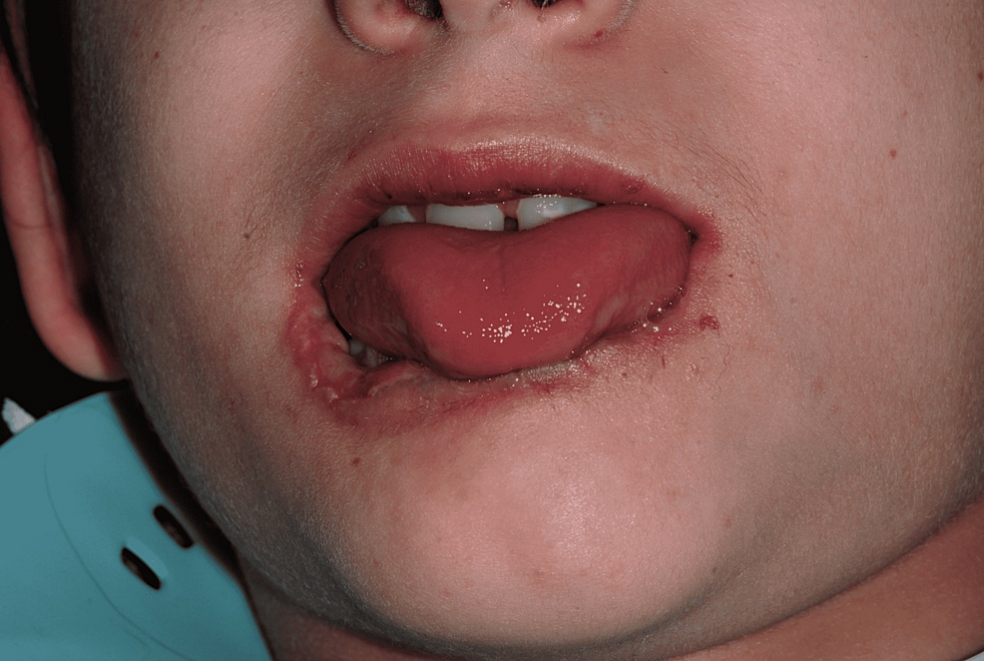
Tongue at the two-month follow-up.

At the two-month follow-up, the biting behavior of the lips and tongue had improved, and the lesions had left a scar of fibrous tissue and regained normal color and texture (Figures 5, 6). The patient presented with significant improvement in the general status, with feeding capacity, adequate nutritional status, and no signs of new self-mutilation. Minimal aesthetic sequelae and no functional limitation were observed after wound healing.

## Discussion

Self-harm behavior may also be present in other syndromes such as Rett’s syndrome, XXY syndrome, autism, mental retardation, Cornelia de Lange, Giles de la Tourette’s syndrome, infections (such as encephalitis), multiple sclerosis, congenital malformations, or congenital insensitivity to pain.

Oral self-injury may be classified as functional or organic. In functional self-injury, the individual deliberately provokes the injury using a method aimed at attracting attention. In cases of organic origin, individuals inflict the injury unconsciously in a compulsive manner and with no specific intent [7].

Currently, there are no guidelines or specific techniques to prevent orofacial self-mutilation. Different intraoral appliances have been designed, including soft heat-formed splints, a posterior splint used to create an anterior open bite, lip bumpers, and mouth guards; however, all with partial success [8]. The possibility of orthognathic surgery to generate an open bite in patients with chronic self-harm of the lip has also been described [9].

Botulinum toxin A injections have shown good results in some case reports as they inhibit the release of acetylcholine from the peripheral nerve endings, resulting in muscle weakness, as well as due to its possible effect on the central nervous system [10].

A proposed solution was the use of acrylic splints; however, it has a low success rate, can cause new lesions or fractures of the splint, which could lead to respiratory risks, and interferes with oral hygiene, increasing the probability of fungal infections.

Sometimes, a radical solution with multiple extractions is needed. This has been shown to produce an enormous reduction in damage to the soft tissues, and some patients in whom dental extraction has been postponed have developed significant deformity of the oral and perioral tissues [11]. The kind of treatment chosen strongly depends on the intensity of the auto-aggressive behavior.

Antiseptics are usually administered to prevent or treat superinfection, and antibiotics have also been used in some cases. It is obligatory to improve oral hygiene to prevent superinfection and facilitate the healing process.

## Conclusions

Most of the scientific literature available on oral self-mutilation is in the form of case reports, making it very difficult to establish clinical guidelines or protocols. Therefore, the therapeutic approach typically needs to be determined for each individual patient, ranging from intraoral devices to multiple tooth extractions in the most severe cases.

In the case described, the oral pathology team decided that the most appropriate therapy was botulinum toxin A injections administered bilaterally into the masseters, along with therapeutic adjustment, which was effective in wound healing and resolution of self-mutilation at the two-month follow-up.
